# No-scalpel vasectomy by electrocauterization in free range rhesus macaques (*Macaca mulatta*)

**Published:** 2012-02-05

**Authors:** V. Kumar, A. Raj

**Affiliations:** 1 *Veterinary Officer, Dhauladhar Nature Park- Gopalpur, Palampur, Distt - Kangra, Himachal Pradesh, India.176059*; 2 *Veterinary Officer, Veterinary Hospital - Deol, Baijnath, Distt - Kangra, Himachal Pradesh, India 176125*

**Keywords:** No-scalpel vasectomy, Electrocauterization, Free range, Rhesus monkey (*Macaca mulatta*)

## Abstract

The objective of the study was to standardize a new method of vasectomy in male rhesus macaques (*Macaca mulatta*). A total of 208 free range male rhesus macaques captured from different locations in Shivalik Hills in a population control programme of the rhesus macaques in India. General anaesthesia was achieved by using a combination of ketamine hydrochloride at 8 mg/kg body weight and xylazine hydrochloride at 2mg/kg body weight intramuscularly in squeeze cage. Surgical procedure of vasectomy was carried out by single-hole no-scalpel technique using a single pre-scrotal skin incision above the median raphae. Spermatic cord was grasped with ringed forceps and was pulled out through the single-hole incision. Vas deferens was separated from the artery-vein complexus and about 3-4 cm portion of vas deferens was resected. Cauterization of both ends of the vas deferens was achieved with electrocautery. The induction time for anaesthesia was 1.40±0.18 min while surgical time for vasectomy was found to be 5.09±0.22 min. Recovery from general anaesthesia was without side-effects after a mean duration of 36.07±1.22 min, whereas the duration of anaesthesia was observed to be 82.27±4.96 min. There were no major complications following the surgery and recovery of animals was smooth. Animals were kept in postoperative care for five days and released at the same capturing site.

## Introduction

Various methods of vasectomy like laser, laparoscopy and open methods are being utilized by various workers in human and animals throughout the world (Harrison *et al.*, 1977; Zhang, 1981; Wildt and Lawler 1985; Sun *et al.*, 1997; Mahalingam *et al.*, 2009). In recent years with the advancement in medical sciences a new approach to expose the vas deferens called no-scalpel vasectomy has received considerable attention because of minimal invasiveness (Marmar *et al.*, 2001). No-scalpel vasectomy, also known as “key-hole” vasectomy (Cook *et al.*, 2007), was developed by Li Shunqiangin, the Sichuan Province of China, in 1974. No-scalpel vasectomy is an advanced technique for performing vasectomy.

No-scalpel technique aims to reduce adverse events, especially hematomas, bleeding, bruising, infection and pain, and to shorten the operating time. At present, there is limited use of no-scalpel vasectomy in captive as well as free range wild animals. The main importance of no-scalpel vasectomy for animals is maintaining the original route of blood supply to gonads thus keeping unaltered all normal physiology of the vasectomized animals. The present study was conducted to utilize the key-hole vasectomy technique in a population control programme of rhesus macaque in order to minimize time for sterilization in male rhesus macaques.

The resulting smaller puncture wound typically has less chance of infection, resulting in faster healing time compared to the larger incision made with a scalpel (Sokal, 2003). The surgical wound created by no-scalpel method usually requires either a single or no suture. This method generally requires more training and skill than the conventional incisional method. The purpose of the present study was to standardize the technique of no-scalpel vasectomy in rhesus macaques.

## Materials and Methods

The study was conducted on 208 male rhesus macaque captured by cage trapping. After capturing, the animals were transported to Monkey Sterilization Centre in transportation cages. Adequate amount of food and water was provided to all animals during transportation. Animals were given rest for one day prior to sterilization.

Animals fasted for 12-15 hrs before the anaesthesia. Each animal was squeezed in squeeze cage of standard size. General anaesthesia was achieved on the basis of estimated body mass. Anesthesia was administered by intramuscular injection of xylazine 2mg/kg body weight and ketamine 8 mg/kg body weight (Troy Laboratories PTY LTD, Australia). Maintenance of anaesthesia was achieved by ketamine only.

Clipping and shaving of the anaesthetized animal was done. Asepsis was achieved by scrubbing of the shaved area with betadine (5%) and followed by 70% alcohol. Animal was positioned in dorsal recumbency (trendelenburg’s position) on a surgical table.

**Fig. 1:**
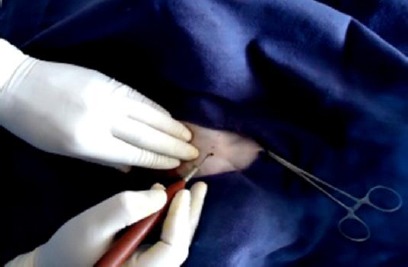
Key-hole incision by cautery pencil.

**Fig.2:**
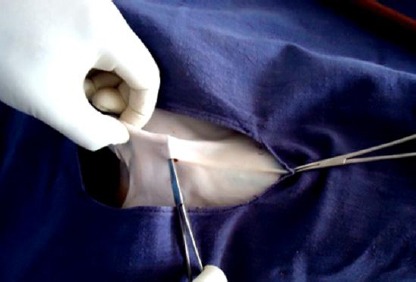
Insertion of ringed forceps and grasping of spermatic cord.

**Fig. 3:**
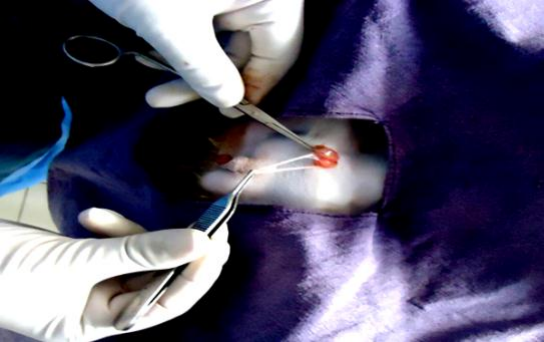
Pulling out spermatic cord and separation of vas deferens.

**Fig.4:**
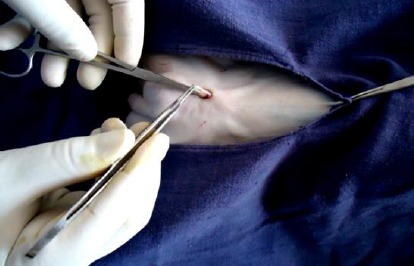
Elevation of vas deferens.

**Fig. 5:**
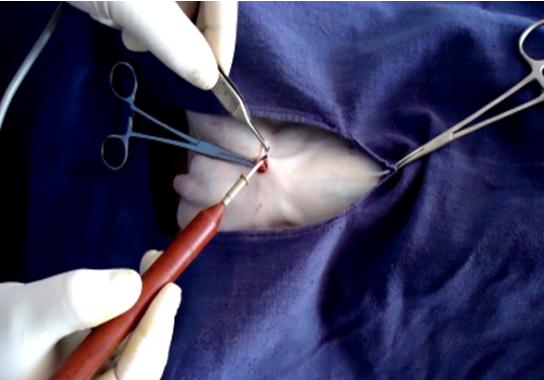
Cutting and cauterization of vas deferens.

Pre-scrotal key-hole incision of about 5 mm was made just above the median raphae with the help of cautery pencil connected to an electrocautery (Fig. 1). The right spermatic cord was felt gently from outside of the skin and the spermatic cord was grasped between the fore finger and thumb of left hands as shown in Fig. 2. A 4-mm ringed forceps with closed tips was passed through the given midline key-hole incision. After piercing through the fascia, the ringed forceps was pushed gently up to the spermatic cord and the spermatic cord was grasped. After grasping the spermatic cord, the ringed forceps was locked. The locked ringed forceps along with the spermatic cord were pulled out through the key-hole incision (Fig. 3). The vaginal tunic was opened by blunt dissection with a rat tooth forceps and vascular and non-vascular portions of the spermatic cords were separated. The artery-vein complexus was stretched with the help of forceps and vas deferens was found attached to it. Then vas deferens was separated gently from the artery-vein complexus. The vas deferens was then elevated with the help of the forceps to make the structure clear (Fig. 4). The vas deferens was transected at two places using an electrocautery (Fig. 5). Both ends of the vas deferens were cauterized at 60 watt current. Vas deferens of about 3-4 cm in length was removed and ends of the vas deferens were allowed to return to the scrotum to their normal position. An identical procedure was performed on the opposite vas deferens through the same keyhole incision. Care is taken to use cautery safely in order to avoid damage to the surrounding tissues. No sutures were applied for closure of the key-hole incision. A Povidine - iodine ointment was applied on the key-hole incision.

In postoperative care, all the animals were given injectable antibiotic and anti-inflammatory. Inj enrofloxacin at 10 mg/kg I/M (Virbac India Ltd) and Inj Melonex (Intas Pharmaceuticals Ltd) at 0.25 mg/kg I/M were administered for three days. Animals were released after five days postoperatively on completion of surgical wound healing.

## Results

The induction of anaesthesia was generally calm and smooth throughout the surgical procedure. The induction time was 1.40±0.18 min while surgical time for vasectomy was 5.09±0.22 min. Physiological parameters such as heart rate (78.08±3.02), respiration rate (32.04±8.16) and rectal temperature (100.28±4.8°F), were measured immediately after anaesthesia. There was slight decrease in respiration rate (28.75 ±2.61) as well as rectal temperature (98.06±1.96°F) which regain normal after 6 hours. The vas deferens was felt with the fingers of the left hand accurately and it was grasped with the ringed forceps held in the right hand. The vas deferens was gradually pulled out and was seen as a light pink-coloured structure. The cauterization of vas deferens was achieved at both testicular as well as prostatic ends effectively. During the cauterization of the cut edge of the vas deferens, the precautions were taken to avoid any injury to the surrounding vascular tissues i.e. artery-vein complexus.

Recovery from general anaesthesia was without side-effects after a mean duration of 36.07±1.22 min, whereas the duration of anaesthesia was observed to be 82.27±4.96 min. There were no major complications except in three cases where little bleeding during exteriorization of artery-vein complexus was observed. All the rhesus macaques recovered within 4-6 hrs after surgery and started taking food and water.

## Discussion

No-scalpel vasectomy has been developed in 1974 in China and then remains confined to the state of China only. It spread to other parts of the world after a long period of time, i.e. more than a decade, in the year 1985.

One of the first steps in vasectomy is to identify the vas deferens so that it can be occluded. The vas deferens located within the spermatic cord can be easily palpated and differentiated from other structures in the cord (spermatic fascia, arteries, and veins), as it appears as thick and soft structure within the spermatic cord.

The vas deferens was held firmly by encircling around with the ring forceps rather than by grasping it with the tips of the ring. The various methods are used by surgeons for the occlusion of the vas deferens out of which vasectomies by electrocautery technique is considered more effective and safer than vas ligation (Barone *et al.*, 2004).

Cautery has been shown to be highly effective than other methods like excision and ligation of vas deferens (Labrecque *et al.*, 2004; Mousavi *et al.*, 2007) and was found to significantly reduce failures compared with ligation and excision with fascial interposition (Sokal *et al.*, 2004).

Advantages of the no-scalpel technique include shorter operative time, less tissue injury, less postoperative swelling and pain and a lower complication rate (Goldstein, 1989; Li *et al.*, 1991). In no-scalpel technique there is need to accurately expose the sheath layer to accomplish appropriate sheath interruption. The no- scalpel vasectomy technique is very simple and also a faster method of vasectomy being practiced nowadays all over the world in human male vasectomy.

A puncture of 4-6 mm was sufficient for exteriorization of the artery-vein complexus. The both ends of the vas deferens were cauterized after cutting of the both ends at a distance of 3-4 cm from each other. The cauterized ends were allowed to return to their position. It was easy to vasectomize both vas deferens from the same key-hole and also it is advantageous as it required even no suture.

In human beings, no-scalpel vasectomy reported less pain during the procedure, early follow-up period than the incisional vasectomy and also reported earlier resumption of routine activity (Skriver *et al.*, 1997; Sokal *et al.*, 1999).

No-scalpel vasectomy can be utilized if a large number of animals are to be vasectomized in a small period of time with less surgical interventions. During vasectomy, smooth muscle relaxation is needed to perform surgery. The combination of xylazine at 2mg/kg and ketamine at 8 mg/kg was sufficient to achieve anaesthesia. Naccarato and Hunter (1979) and Kumar *et al*. (2011) have also used xylazine and ketamine for effective anaesthesia in rhesus macaques. The complications reported in three cases were treated. All animals recovered smoothly after anaesthesia and survived the surgery. All animals started taking food and water normally.

It is concluded that in a mass population control programme of rhesus macaque, this procedure proved to be a safe, effective with less bleeding, hematoma, infection and shorter operation time than the conventional incision technique. This technique can be effectively utilized in the vasectomy of the male rhesus macaques.
